# Rare Variants in Novel Candidate Genes Associated With Nonsyndromic Patent Ductus Arteriosus Identified With Whole-Exome Sequencing

**DOI:** 10.3389/fgene.2022.921925

**Published:** 2022-06-06

**Authors:** Ying Gao, Dan Wu, Bo Chen, Yinghui Chen, Qi Zhang, Pengjun Zhao

**Affiliations:** ^1^ Department of Pediatric, Shidong Hospital, Shanghai, China; ^2^ Department of Cardiothoracic Surgery, School of Medicine, Heart Center, Shanghai Children’s Medical Center, School of Medicine, Shanghai Jiao Tong University, Shanghai, China; ^3^ Department of Pediatric Cardiology, Xin Hua Hospital, School of Medicine, Shanghai Jiao Tong University, Shanghai, China

**Keywords:** congenital heart defects, patent ductus arteriosus, whole-exome sequencing, rare variants, single-nucleotide polymorphism

## Abstract

**Background:** Patent ductus arteriosus (PDA) is one of the most common congenital heart defects causing pulmonary hypertension, infective endocarditis, and even death. The important role of genetics in determining spontaneous ductal closure has been well-established. However, as many of the identified variants are rare, thorough identification of the associated genetic factors is necessary to further explore the genetic etiology of PDA.

**Methods:** We performed whole-exome sequencing (WES) on 39 isolated nonsyndromic PDA patients and 100 healthy controls. Rare variants and novel genes were identified through bioinformatic filtering strategies. The expression patterns of candidate genes were explored in human embryo heart samples.

**Results:** Eighteen rare damaging variants of six novel PDA-associated genes (*SOX8*, *NES*, *CDH2*, *ANK3*, *EIF4G1*, and *HIPK1*) were newly identified, which were highly expressed in human embryo hearts.

**Conclusions:** WES is an efficient diagnostic tool for exploring the genetic pathogenesis of PDA. These findings contribute new insights into the molecular basis of PDA and may inform further studies on genetic risk factors for congenital heart defects.

## Introduction

The ductus arteriosus (DA) is a normal fetal structure that connects the pulmonary artery and descending aorta to maintain blood circulation during the fetal period (Benitz, W. E. et al., 2016). From the perspective of cardiac development, the DA functionally shuts down 15 h after birth in healthy, full-term infants (Crockett, S. L. et al., 2019). This process involves abrupt contraction of the muscular wall of the DA, which is associated with a proper balance among neurohumoral factors. An increase in the levels of contractile elements, such as peroxidase O2 and endothelin-1, and decrease in the levels of relaxants, such as prostaglandin E2 and nitric oxide, are the main events causing closure of the DA (Crockett, S. L. et al., 2019). Neural crest-derived cells migrate into the subendothelial space under the action of these hormones and transform into vascular smooth muscle cells (VSMCs). With contraction of the medial membrane and circular muscle in the DA, the lumen is shortened and finally closed (Li, N. et al., 2016). However, the maintenance of DA patency after birth has a pathological effect (Benitz, W. E. et al., 2016).

Failure of the DA to close after birth is termed patent DA (PDA), which is one of the most common heart defects, affecting approximately 1 in 2000 full-term infants and 8 in 1000 premature infants (Hoffman, J. I. et al., 2002). Persistent ductal shunting may lead to pulmonary overcirculation and induce systemic hypoperfusion, thereby increasing the risk of pulmonary hypertension, infective endocarditis, heart failure, and even death (Mitra, S. et al., 2018). However, its etiology and pathogenesis remain unclear.

PDA has both inherited and acquired causes. The preliminary understanding of the genetic mechanism of PDA was based on the studies in patients with syndrome. Previous studies have confirmed the association of several chromosomal syndromes, including Turner (45, XO), Kartagener, and Klinefelter (47, XXY), with PDA (Groth, K. A. et al., 2013; Gravholt, C. H. et al., 2019; Yang, D. et al., 2019). In addition to chromosomal rearrangements, a single gene mutation can also cause syndromic PDA, including Noonan (*PTPN11* mutation), Holt-Oram (*TBX5* mutation), and char (*TFAP2B* mutation) syndrome (Satoda, M. et al., 2000; Pannone, L. et al., 2017; Vanlerberghe, C. et al., 2019). However, the genetic mechanism of nonsyndromic PDA (isolated findings without other abnormalities) remains unclear. Rare damaging mutations in *MYH11* and *TFAP2B* were detected in several isolated nonsyndromic PDA patients (Harakalova, M. et al., 2013). Erdogan et al. (Erdogan, F. et al., 2008) performed an array comparative genome hybridization analysis of 105 patients with congenital heart defects and identified a 1.92 Mb deletion of chromosome 1q21.1 (CJA5) in a PDA patient. Genetic determinants of nonsyndromic PDA is still unknown.

Therefore, in this study, we recruited 39 unrelated nonsyndromic PDA patients and 100 healthy children for WES. Using a series of bioinformatics filtering steps, we identified 18 rare damaging variants in six candidate PDA-associated genes (*SOX8*, *NES*, *CDH2*, *ANK3*, *EIF4G1*, and *HIPK1*). Notably, these candidate genes were also highly expressed in human embryonic hearts. This identification of new pathogenic genes could help to elucidate the detailed underlying mechanism of PDA and promote further experimental analyses.

## Material and Methods

### Patients and Consent

Thirty-nine isolated nonsyndromic PDA patients of Han Chinese ethnicity and 100 healthy children (aged between 2 months and 13 years) were recruited from Xinhua Hospital affiliated with Shanghai Jiao Tong University (Shanghai, China). The structural heart phenotypes of all participants were assessed using echocardiography or cardiac catheterization. A diagnosis of PDA was made in the patient group by cardiac catheterization or surgery. Patients with a history of complex congenital heart disease were excluded from the study. The study protocol and ethics were approved by the Medical Ethics Committee of Xinhua Hospital. Informed consent was obtained from the parents of all participants. The study was conducted in accordance with the Declaration of Helsinki and the International Ethical Guidelines for Health-Related Research Involving Humans.

### DNA Extraction and Whole-Exome Sequencing

The genomic DNA of all participants was extracted from blood samples using QIAamp DNA Blood Mini Kit (QIAGEN, Germany). DNA samples were stored at –80°C until further use. Genomic DNA was eluted, purified, amplified by ligation-mediated polymerase chain reaction, and then subjected to DNA sequencing on an Illumina platform. The target depth of the DNA sequencing was x100. Qualified DNA samples from the PDA and control groups were subjected to WES to detect rare variations. Read quality was checked using Fastp software(Chen, S. et al., 2018) and raw sequence data were aligned to human genome (human_glk_v37) using BWA (v0.7.12-r1039). Duplicated and low-quality reads (Per base sequence quality <20) were removed by using Picard software (https://broadinstitute.github.io/picard). Alignment quality was assessed using qualimap software (Okonechnikov, K. et al., 2016).

### Single-Nucleotide Polymorphism Identification and Quality Filtering

Single nucleotide polymorphisms (SNPs) account for much of the phenotypic diversity among individuals.

**FIGURE 1:**
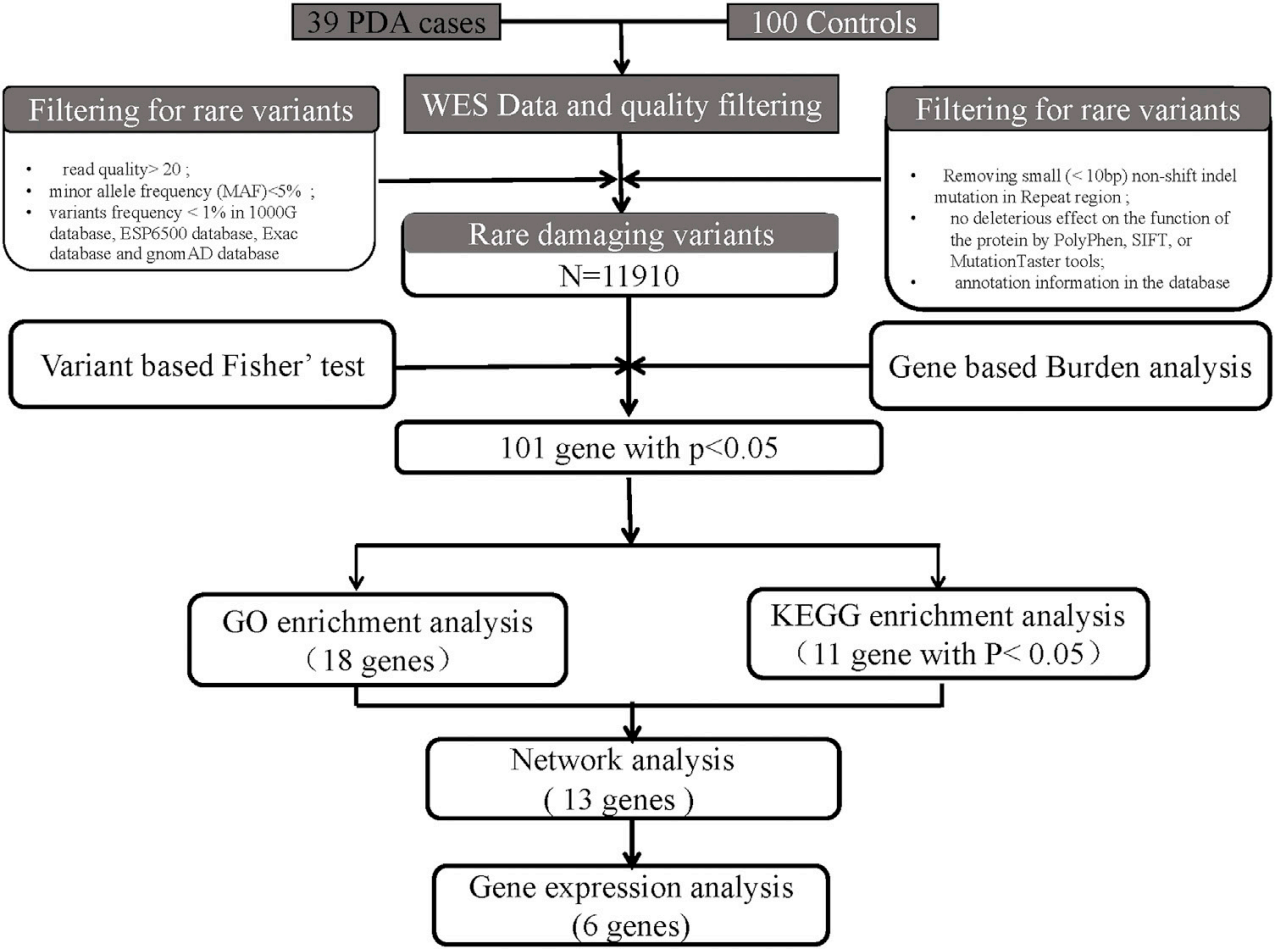
Bioinformatics filtering strategy workflow for the candidate genes. Through a series of filtering methods, we finally identified 6 candidate genes. The potentially damaging variants in candidate genes were subjected to validation *via* human embryonic heart expression analysis.

SNPs and insertions/deletions were detected using the HaplotypeCaller module of GATK4 software (Mckenna, A. et al., 2010), based on sequence alignment of the clinical samples to the reference genome. Before detection, we recalibrated the base qualities using the BaseRecalibrator module of GATK4 software (Mckenna, A. et al., 2010) to improve variant detection accuracy based on the quality with a depth (QD) criterion >2. The resulting BAM files were then sorted, indexed, and processed using base quality score recalibration (Okonechnikov, K. et al., 2016). The GATK HaplotypeCaller module was then used for variant calling. We used ANNOVAR53 (Wang, K. et al., 2010) to annotate the variants for functional and population frequency information with the 1000 Genomes (Clarke, L. et al., 2012), Refseq (O’leary, N. A. et al., 2016), ExAC (Karczewski, K. J. et al., 2017), ESP6500 (Liang, Y. et al., 2019), gnomAD, SIFT (Flanagan, S. E. et al., 2010), clinvar (Landrum, M. J. et al., 2020), PolyPhen (Flanagan, S. E. et al., 2010), MutationTaster (Steinhaus, R. et al., 2021), COSMIC (Forbes, S. A. et al., 2011), gwasCatalog, and OMIM databases (Amberger, J. S. et al., 2017). All potentially damaging variants of the candidate genes were classified into five groups: pathogenic, likely pathogenic, variant of uncertain significance, likely benign, and benign (Richards, S. et al., 2015). Finally, the rare damaging variants were filtered according to the American College of Medical Genetics criteria guidelines (Figure 1).

### Variant Filtering Based on Fisher’s Exact Test and Burden Analysis

The difference in allele frequency for each SNP between cases and controls was compared using the Fisher’s exact test with R statistical software packages; a *p*-value < 0.05 was considered statistically significant. Subsequently, we aggregated the SNP data based on gene expression levels and conducted a gene-based burden analysis to increase statistical power. Candidate pathogenic genes were filtered based on the results of burden analysis according to the following criteria: 1) *p*-value or false-discovery rate (FDR) < 0.05, 2) hit for at least one variant in three cases, and 3) not found in any sample of the control group. We then prioritized genes based on the *p* value of Fisher’s exact test and burden analysis.

### Functional Enrichment and Network Analysis

To further filter the candidate genes associated with PDA, we performed functional enrichment analysis to identify the functions of candidate genes identified through the aforementioned filtering steps. Pathway analysis of the candidate gene profiling results was performed using Gene Ontology (GO; version 30.10.2017) and Kyoto Encyclopedia of Genes and Genomes (KEGG) pathway (http://www.genome.jp/kegg/pathway.html) mapping within the web-based tool Database for Annotation, Visualization, and Integrated Discovery (Gene Ontology, C. 2015; Kanehisa, M. et al., 2017). GO terms represent a network of biological processes that overlap in space and are clustered according to their relationships (Gene Ontology, C. 2015). The threshold was set to an adjusted *p*-value < 0.05. In addition, we prioritized these genes based on functional enrichment analysis. Furthermore, to detect the relationship between the candidate genes and known disease-causing genes, we constructed the protein–protein interaction (PPI) network (Brohee, S. et al., 2008) using Cytoscape software based on the STRING database.

### Tissue Collection and Expression Detection

In addition to the genes prioritized using the steps described above, we further prioritized genes according to their expression levels in the human embryonic heart. Previous studies have divided eight embryonic weeks (56 days) into 23 internationally accepted Carnegie stages (O’rahilly, R. 1987). To further investigate the potential function of our candidate genes, human embryonic hearts in different Carnegie stages (S10–S16) were collected after medical termination of pregnancy from patients at Xinhua Hospital. RNA was extracted and purified using the Experion automated gel electrophoresis system and RNeasy MinElute Cleanup Kit. The expression patterns of candidate genes were subsequently detected using the Affymetrix HTA 2.0 microarray.

## Results

### Population

**FIGURE 2:**
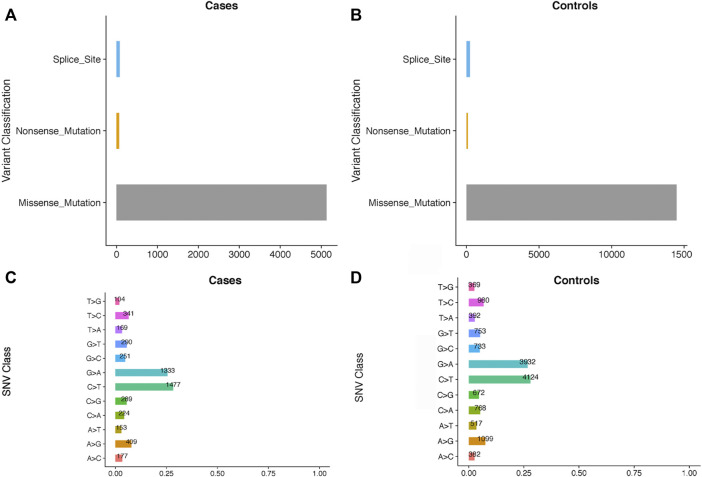
The comparisons of the rare damaging variants between the PDA and control groups. The number of variants in each variant classification and SNV class between cases and controls are presented in **(A–D)**, respectively.

Among the 39 patients, 28% had common cardiac defects, including atrial septal defect (*n* = 7), ventricular septal defect (*n* = 2), and others (*n* = 2) (Table 1). All subjects were born at full term, and no other major cardiac structural abnormalities or developmental syndromes were identified. WES, with an average depth of coverage of approximately x105 per base, identified 411,344 single-nucleotide variants and 23,101 insertions/deletions across the genome. Through a series of filtering strategies (see Figure 1), rare damaging variants were screened with a threshold of 0.5% minor allele frequency. As illustrated in Figure 2, we found more rare damaging variants in the PDA group than in the control group, including splice-site, nonsense, and missense mutations. Consistently, the C > T and G > A substitutions accounted for the majority of single-base mutations compared with other types (Figure 2). Based on these mutations, we adopted a bioinformatics filtering strategy to identify candidate genes associated with PDA.

**TABLE 1:** Characteristics of 39 PDA patients.

Patients characteristics	Numbers
Age (year)	2.92 ± 2.44
Male-to-female ration (%)	62%
BMI (kg/m2)	16.58 ± 4.34
PDA size (mm)	2.87 ± 1.68
Birth weight (kg)	2.96 ± 0.73
Gestational age (week)	39.04 ± 1.46
Associated cardiac defect n (%)	
VSD n (%)	2 (5%)
ASD n (%)	7 (18%)
Others n (%)	2 (5%)

All values are expressed as mean ± SD or n (%)

### Variants Identified Based on Fisher’s Exact Test

**FIGURE 3:**
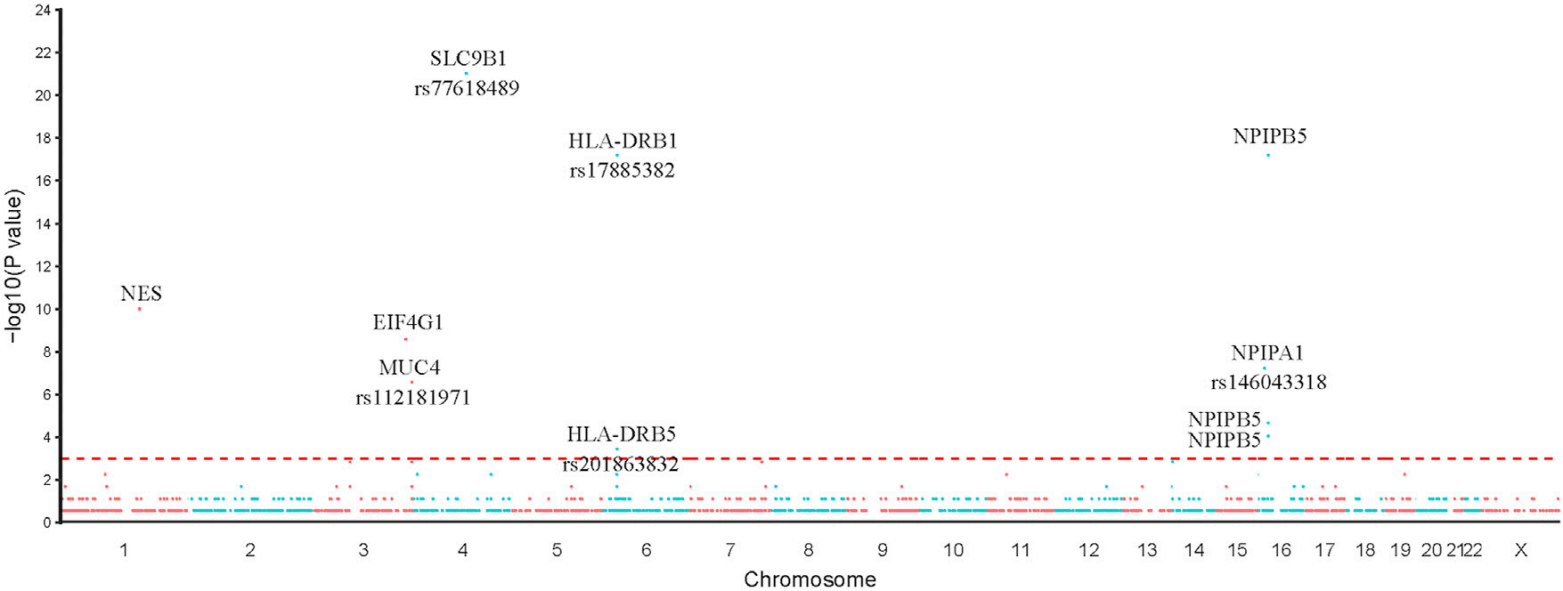
Single SNP allele frequency and genotype frequency *p*-values were obtained using the fisher exact test. X-axis represents the position of each snp (represented in circles) on human chromosome, Y-axis is the–log *p*-value of Fisher Exact test. Top 10 variants in our study were represented in the figure.

Based on the results of Fisher’s exact test, we identified 44 variants that were more frequently detected in the PDA group than in the control group (FDR <0.05, *p* < 0.05), as presented in Table 2 (*p* < 0.01). We then prioritized these variants based on the *p*-value; the top 10 variants with statistical significance are shown in Figure 3. Notably, we found that the SNPs rs103826685 and rs32552095 located in *SLC9B1* and *HLA-DRB1*, respectively, had the most significantly different frequencies between the patient and control groups (*p* < 0.0001).

**TABLE 2:** SNP filtering Based on Fisher Exact Test.

Chromosome	Gene	Mutation position	Mutation type	*p*-value
1	LRRC8C	90179703	T > G	0.006
1	NES	156640657	A > C	0.000
11	LRRC4C	40136434	C > T	0.006
14	SLC7A8	23612372	T > G	0.001
16	SOX8	1034733	A > C	0.006
16	NPIPA1	15045634	T > C	0.000
16	NPIPB5	22545658	A > C	0.000
16	NPIPB5	22546505	G > T	0.000
16	NPIPB5	22546506	A > C	0.000
19	MAP3K10	40719910	C > G	0.006
3	ZNF717	75786264	G > T	0.001
3	EIF4G1	184033621	G > C	0.000
3	MUC4	195506722	G > A	0.001
3	MUC4	195506723	T > G	0.001
3	MUC4	195514174	G > T	0.000
4	USP17L20	9217567	C > A	0.006
4	USP17L17	9246041	C > A	0.006
4	SLC9B1	103826685	T > A	0.000
4	LRBA	151770608	A > C	0.006
6	VARS	31746821	G > A	0.006
6	HLA-DRB5	32487344	T > C	0.000
6	HLA-DRB1	32552095	C > T	0.000
7	TCAF2	143400090	G > A	0.001

### Candidate Genes Identified Based on Burden Analysis

**FIGURE 4:**
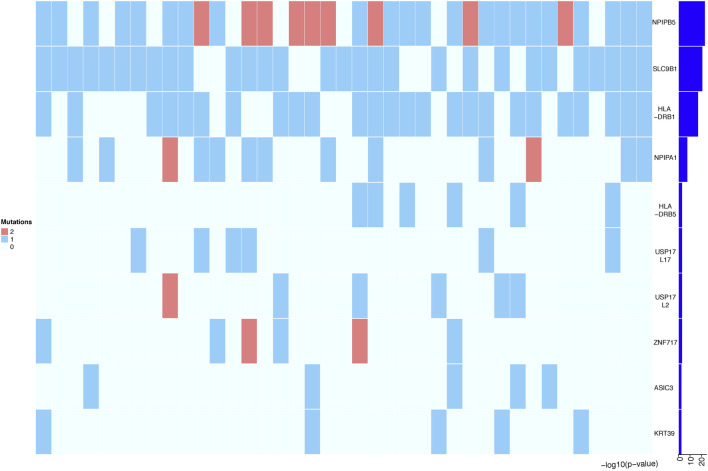
Heatmap representing the top 10 genes identified in Burden analysis. Heatmap that shows the mutational burden (*p*-value< 0.05) of the top 10 gene based on gene-based burden analysis in PDA patients. The heatmap was generated by using R package, the mutation values were normalized per gene over all PDA samples. Each box in the heatmap represent a single variant in a case, with the dark red indicating high gene mutation ration in gene-based Burden analysis.

To further increase statistical power, we aggregated the SNP data at the gene level and performed burden analysis. Under a significance threshold of 0.05, we observed 57 genes with potential pathogenicity as candidate PDA-associated genes (Table 3 (*p* < 0.01)). We then prioritized these genes based on the *p*-value from the burden analysis; the top 10 genes with statistical significance are displayed as a heatmap in Figure 4. The top three genes with high confidence were *NPIPB5*, *SLC9B1*, and *HLA-DRB1*. Notably, *SLC9B1* and *HLA-DRB1* were also in the top significant genes based on Fisher’s exact test.

**TABLE 3:** Gene filtering based on Burden analysis.

Gene	Case mutation	Case normal	Control mutation	Control normal	*p*-value
ASIC3	5	34	0	100	0.001
CFAP45	4	35	0	100	0.006
CYP21A2	4	35	0	100	0.006
EVI5	4	35	0	100	0.006
HIPK1	4	35	0	100	0.006
HLA-DRB1	25	14	0	100	0.000
HLA-DRB5	6	33	0	100	0.000
KRT39	5	34	0	100	0.001
LRRC4C	4	35	0	100	0.006
MAP3K10	4	35	0	100	0.006
NPIPA1	13	26	0	100	0.000
NPIPB5	31	8	0	100	0.000
POTEE	5	34	0	100	0.001
SLC9B1	29	10	0	100	0.000
SLX4	4	35	0	100	0.006
SOX8	4	35	0	100	0.006
TBC1D3F	4	35	0	100	0.006
TCAF2	5	34	0	100	0.001
USP17L11	5	34	0	100	0.001
USP17L17	6	33	0	100	0.000
USP17L18	5	34	0	100	0.001
USP17L2	6	33	0	100	0.000
USP17L20	5	34	0	100	0.001
VARS	4	35	0	100	0.006
ZNF717	6	33	0	100	0.000

### Functional Analysis

**FIGURE 5:**
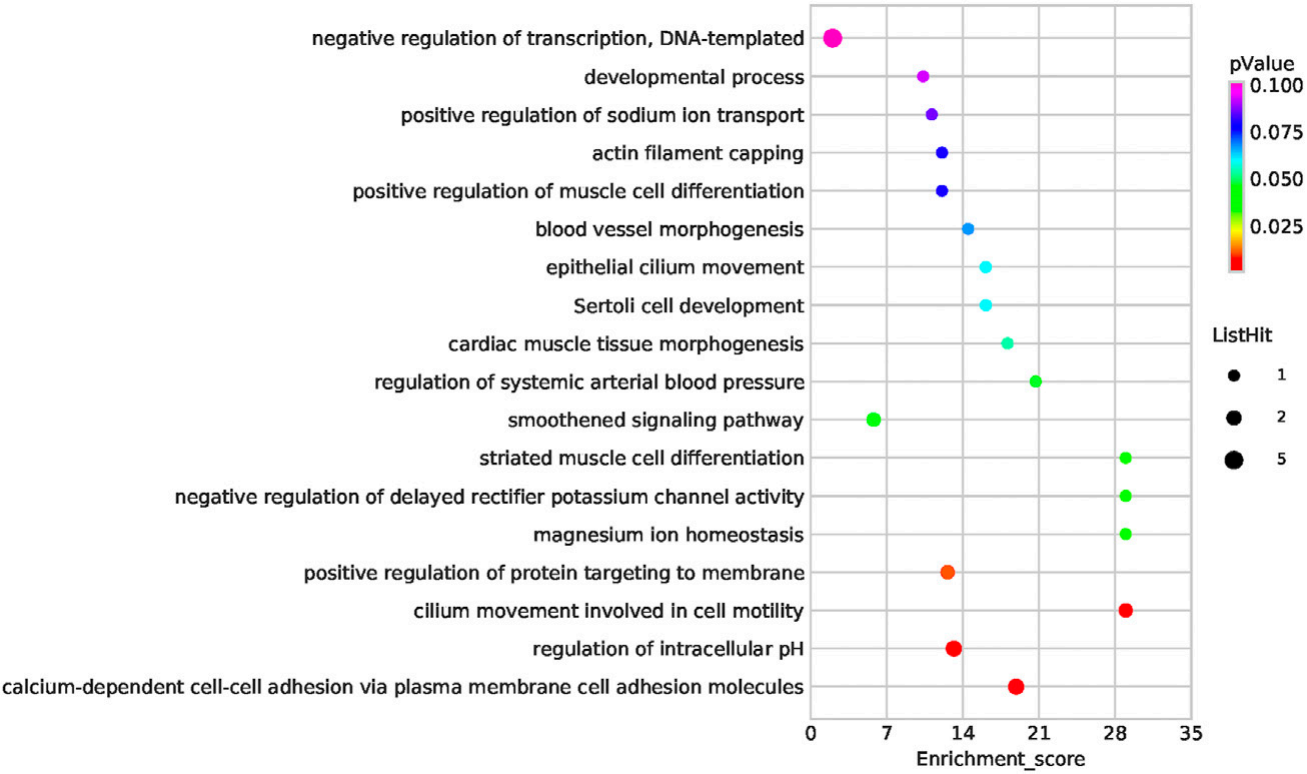
Bubble plot of the GO analysis. Bubble plot summarizing enrichment for the most significant biological process GO terms associated to differentially expressed genes. The bubble size indicates the frequency of the GO term, while the color indicates the *p*-value.

**FIGURE 6:**
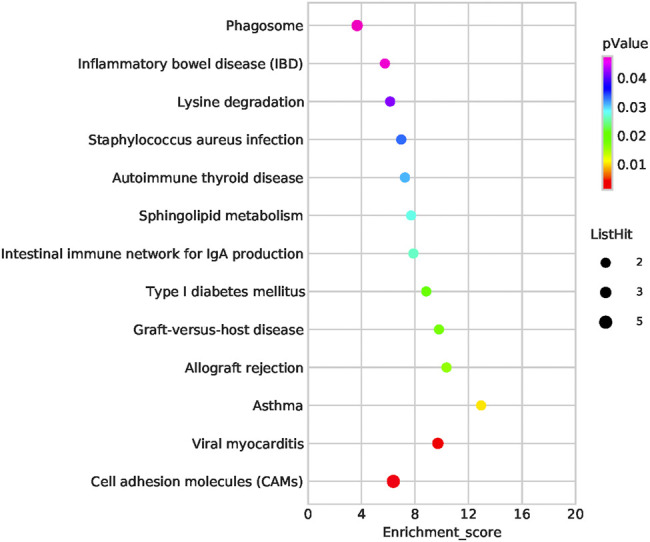
Bubble plot of the KEGG pathway analysis. The representative enriched pathways shown by KEGG analysis. The bubble size indicates the frequency of the KEGG term, while the color indicates the *p*-value.

Functional enrichment analysis of the 101 candidate differentially expressed genes identified through Fisher’s exact test and burden analysis revealed that the main enriched GO terms in the upregulated gene set were thiol-dependent ubiquitinyl hydrolase activity (TermID: GO:0036459), peptide antigen binding (TermID: GO:0042605), and ubiquitin-dependent protein catabolic process (TermID: GO:0006511). Particular focus was placed on terms representing prostaglandin, apoptosis, and heart development (Figure 5). Moreover, KEGG analysis of the direct gene targets in PDA patients revealed enrichment in pathways related to cell adhesion molecules (TermID: path: hsa04514, *p* < 0.001), viral myocarditis (TermID: path: hsa05416, *p* = 0.0035), and asthma (TermID: path: hsa05310, *p* = 0.01; Figure 6). Based on functional enrichment analysis, 29 pathway genes related to cardiovascular development were screened.

### Network Analysis

**FIGURE 7:**
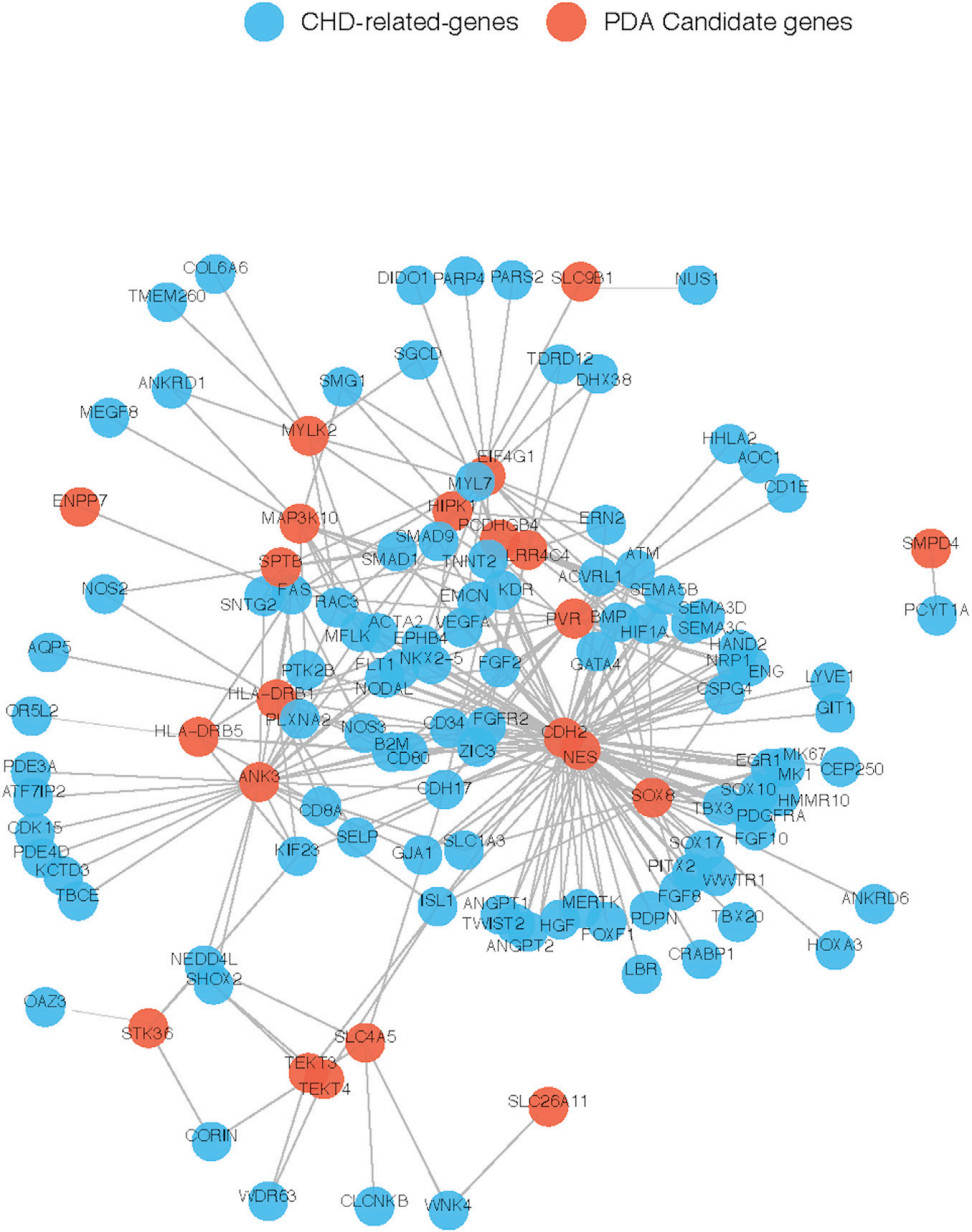
Interaction between our candidate genes and known CHD-related genes. PPI network was generated by Cytoscape software and our candidate pathogenic genes and the known CHD-related genes were uploaded in STRING database. Each node represents one gene, and each edge represents the protein-protein interaction collected from BioGRID.

**FIGURE 8:**
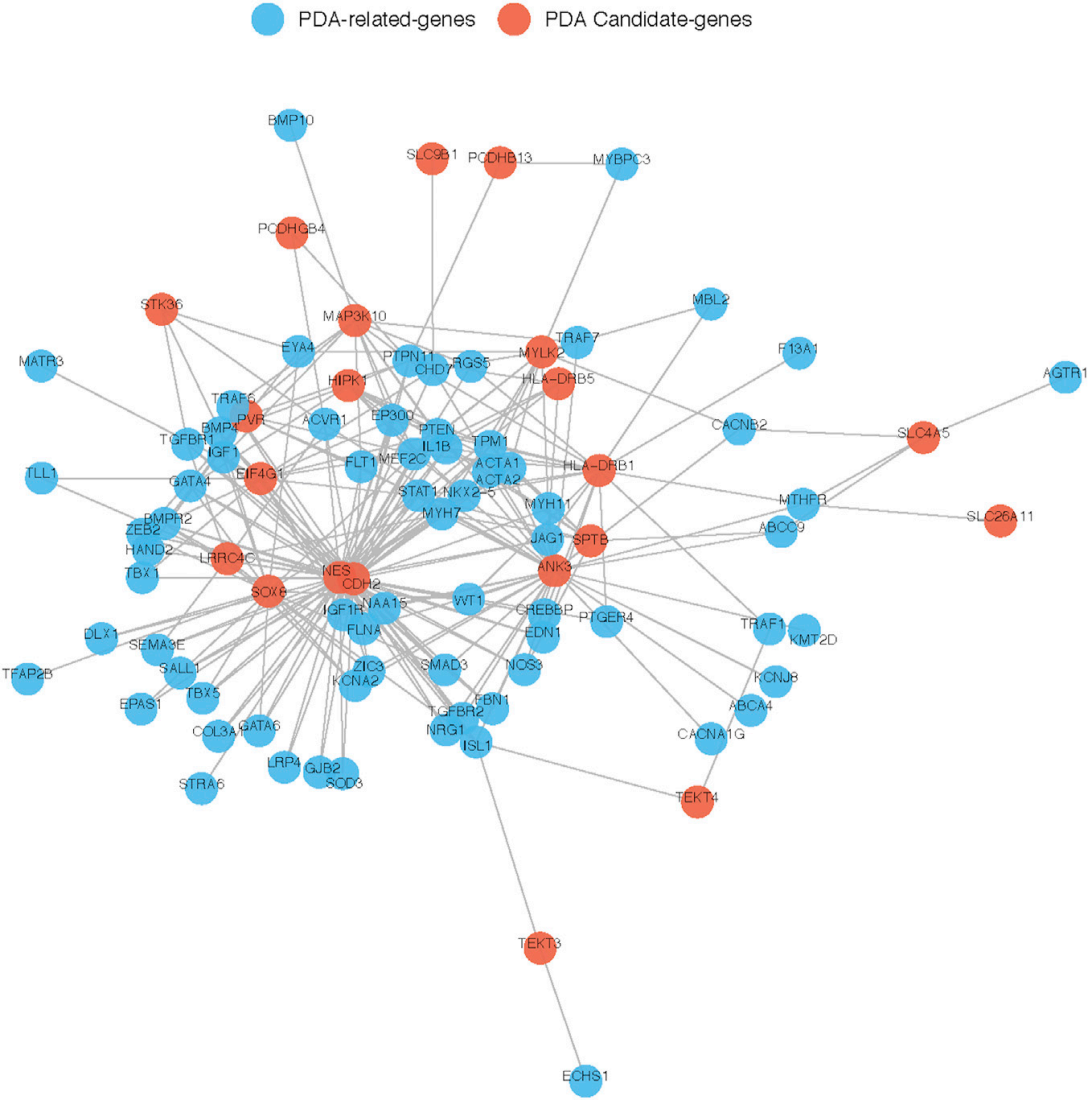
Interaction between our candidate genes and known PDA-related genes. PPI network was generated by Cytoscape software and Our candidate pathogenic genes and the known CHD-related genes were uploaded in STRING database. Each node represents one gene, and each edge represents the protein-protein interaction collected from BioGRID.

To further explore their roles, the 29 candidate genes were mapped to construct a PPI network along with 240 known pathogenic genes involved in cardiovascular development (Supplementary Table S1). The 240 known genes from the literature were divided into two groups related to cardiovascular development and PDA, respectively. In the network, the candidate genes *NES* and *CDH2* showed the most direct and strongest relationship with known pathogenic genes in both groups. Moreover, CDH2 and NES had the highest molecular weights and were located at the center of the PPI network (Figures 7, 8). Therefore, based on the degree of correlation, we screened out 11 candidate genes for final verification.

### Detection of Candidate Gene Expression in the Human Embryonic Heart

**FIGURE 9:**
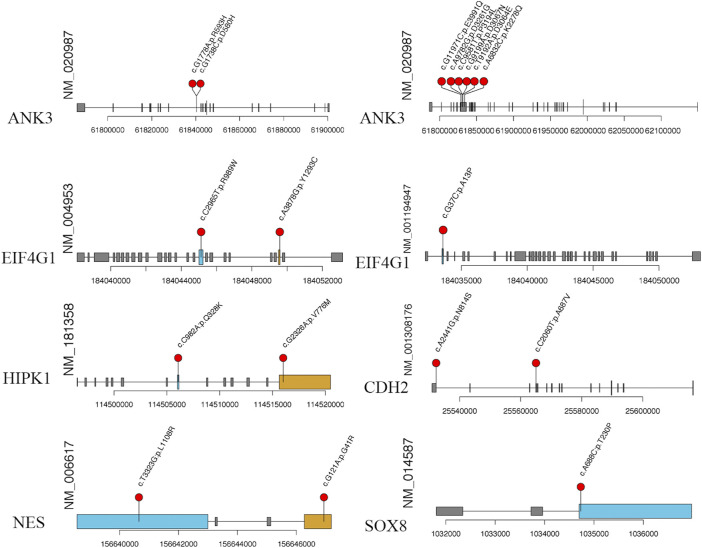
The specific amino acid sites of variants of our candidate gene. The red balls represent the location of rare variant on the encoded proteins or protein domains.

**FIGURE 10:**
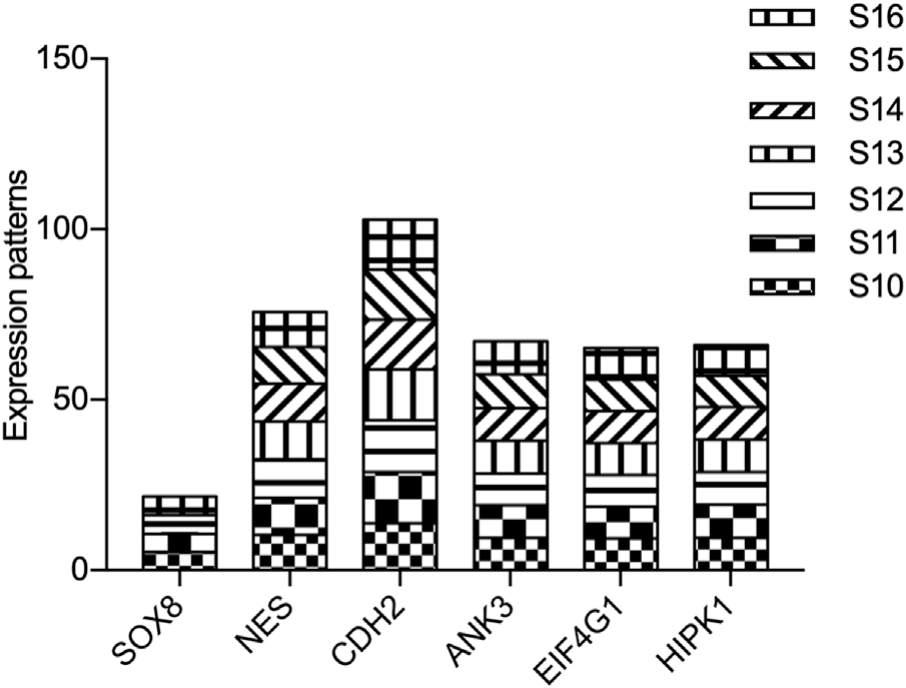
Expression of candidate genes in human embryonic heart. The expression patterns of candidate genes in human embryonic heart at different stages of S10 to S16 were analyzed by microarray. X-axis represents the different stages of human embryonic heart, while the Y-axis indicates the level of gene expression.

To further investigate the potential function of our candidate genes, we detected the expression levels of the 11 screened out genes in human embryonic hearts at different Carnegie stages. After prioritizing the candidate genes based on expression levels, the final six pathogenic genes (*SOX8*, *NES*, *CDH2*, *ANK3*, *EIF4G1*, and *HIPK1*) were identified (Figure 9). Among them, *CDH2* was the most highly expressed in the embryonic heart (Figure 10).

## Discussion

The underlying molecular genetic mechanisms of PDA remain largely unknown as one of the most common congenital heart defects. In this study, we explored the clinical characteristics of 39 PDA patients and 100 healthy controls by performing WES to identify rare variants and candidate PDA-associated genes. Through a series of bioinformatic filtering strategies, we prioritized the candidate genes *via* Fisher’s exact test, mutation burden analysis, gene network construction, and expression levels in embryonic hearts. Finally, we identified 18 rare damaging variants in six novel candidate genes (*SOX8*, *NES*, *CDH2*, *ANK3*, *EIF4G1*, and *HIPK1*) associated with PDA. Among these, *CDH2* was highly expressed in the human embryonic heart and appears to be the most important candidate gene identified in our study.

*CDH2* encodes N-cadherin, a member of a protein family regulating cadherin-mediated cell–cell adhesion in multiple tissues. The structure comprises a single transmembrane domain, cytoplasmic domain, and five conserved extracellular cadherin domains (ECI–V) (Alimperti, S. et al., 2015). We found two variants (rs25565020 and rs25532304) in *CDH2* in four patients with PDA. In addition, CDH2 had the highest molecular weight and was located at the center of the PPI network, both among known CHD- and PDA-related genes. Further investigation showed that *CDH2* is highly expressed in human embryonic hearts. Previous studies in mice have also noted the importance of CDH2 in the proper development of the heart, brain, and skeletal structures (Radice, G. L. et al., 1997). Moreover, genetic analyses in zebrafish revealed that mutation in the EC-I or EC-IV domains of *cdh2* play important role in embryonic development (Masai, I. et al., 2003). Mayosi, B. M. et al. (2017) used WES to detect novel rare variants in patients with arrhythmogenic cardiomyopathy and found that *CDH2* mutation changes the conserved amino acids of CDH2 protein. Since the relationship between *CDH2* and PDA is unclear, additional studies are needed to determine how genetic perturbations of *CDH2* contribute to PDA.

In our study, 16 patients (42%) had the same variant (rs156646936) in *NES*. In the network analysis, we observed a strong correlation between *NES* and known pathogenic genes. NES belongs to the human tissue kallikrein family of secreted serine proteases (Luo, L. et al., 1998), which play an important role in carcinogenesis, including in breast, prostate, and testicular cancers, and leukemia (Luo, L. Y. et al., 2001). Further experimental evidence suggests that the function of *NES* as a tumor suppressor may be achieved by hypermethylation of the CpG islands (Li, B. et al., 2001). However, this is the first report of *NES* mutations in PDA. ANK3 is a member of the ankyrin family, which is expressed in several different isoforms in many tissues. ANK3 plays key roles in cell motility, activation, proliferation, contact, and the maintenance of specialized membrane domains. In our study, eight patients (10%) had variants in *ANK3*. *ANK3* variants have previously been associated with schizophrenia, autism, epilepsy, and intellectual disability (Leussis, M. P. et al., 2013; Wirgenes, K. V. et al., 2014). Studies from knockout mouse models have revealed that loss of ANK3 function leads to defects in cardiac calcium handling and arrhythmias (Mohler, P. J. et al., 2004). Although the roles of *NES* and *ANK3* in the pathogenesis of PDA are supported by bioinformatic analyses, our study was limited by the lack of experimental evidence to validate the deleteriousness of the variants.

*EIF4G1* encodes a protein, that is, a component of the multi-subunit protein complex EIF4F. EIF4G plays a crucial role in translation initiation and serves as a scaffolding protein that binds several initiation factors (the cap-binding protein eIF4E, the RNA helicase eIF4A, and eIF3) (Haimov, O. et al., 2018). In our study, 15 patients had three types of variants in *EIF4G1*, and the same variant (rs184033621) was detected in 14 patients. EIF4G1 modulates the proliferation, apoptosis, and angiogenesis of most tumor types by limiting steps during the initiation phase of protein synthesis and interacting with ubiquitin-specific protease 10 (USP10) (Cao, Y. et al., 2016). Moreover, EIF4G1 phosphorylation specifically activates the PKC-Ras-ERK signaling pathway, which is involved in the control of cell growth and proliferation (Dobrikov, M. et al., 2011). Diseases associated with EIF4G1 include Parkinson’s disease, nonsmall cell lung carcinoma, and prostate cancer (Cao, Y. et al., 2016). Although the relationship between EIF4G1 and cardiovascular development remains unknown, our results suggest that EIF4G1 might be potentially pathogenic in terms of PDA.

HIPK1 belongs to the Ser/Thr family of protein kinases as part of the HIPK subfamily. HIPK1 is related to pathways involved in the regulation of TP53 activity and cardiac conduction. The homeodomain-interacting protein kinases HIPK1 and HIPK2 play key roles in embryonic development by regulating transforming growth factor β-dependent angiogenesis (Aikawa, Y. et al., 2006; Shang, Y. et al., 2013). HIPK1 loss-of-function conditional knockout mice exhibit defects in primitive/definitive hematopoiesis, vasculogenesis, angiogenesis, and neural tube closure (Shang, Y. et al., 2013). In addition, HIPK1 can interact with homeobox proteins and other transcription factors to regulate various biological processes, including signal transduction, apoptosis, embryonic development, and retinal vascular dysfunction (Aikawa, Y. et al., 2006). In our study, only two *HIPK1* variants (rs114516009 and rs114506069) were detected in four individuals with PDA; these are novel variants that have not been reported previously. Further investigation showed that *HIPK1* is highly expressed in human embryonic hearts. However, additional experiments are needed to determine the genetic mechanism by which *HIPK1* contributes to PDA.

SOX8 is a member of the SRY-related HMG-box (SOX) family of transcription factors, which are involved in the regulation of embryonic development and in determining cell fate (Haseeb, A. et al., 2019). In our study, the same rare variant (rs1034733) was detected in three patients with PDA. SOX8 expression is essential in the developing heart, which correlates with heart septation and differentiation of the connective tissue of the valve leaflets (Montero, J. A. et al., 2002). Moreover, a previous study revealed that SOX8 overexpression might be associated with hypoxia-induced cell injury by activating the PI3K/AKT/mTOR and MAPK pathways (Gong, L. C. et al., 2017). Interestingly, DA closure after birth is closely related to the blood oxygenation level, and hypoxia can lead to an increase in endogenous PGE2 release, which directly leads to opening of the DA (Benitz, W. E. et al., 2016). Therefore, *SOX8* may be a novel candidate gene involved in the pathogenesis of PDA.

In conclusion, through a series of bioinformatics filtering steps, we identified 18 rare damaging variants in six novel candidate genes (*SOX8*, *NES*, *CDH2*, *ANK3*, *EIF4G1*, and *HIPK1*) associated with PDA. The discovery of these genes opens up a new field for genetic research on PDA and provides new ideas for understanding the pathogenesis of PDA. Nevertheless, our study has some limitations. The lack of parental samples and the small sample size limited our ability to identify the detailed genetic background of PDA. Thus, more fundamental research is needed to determine candidate genes that contribute to PDA. We hope to confirm these findings with larger sample sizes.

## Data Availability

The datasets presented in this study can be found in online repositories. The names of the repository/repositories and accession number(s) can be found below: https://www.ncbi.nlm.nih.gov/, SRP288538.

## References

[B1] Aikawa Y. Nguyen L. A. Isono K. Takakura N. Tagata Y. Schmitz M. L. (2006). Roles of HIPK1 and HIPK2 in AML1- and P300-dependent Transcription, Hematopoiesis and Blood Vessel Formation. EMBO J. 25 (17), 3955–3965. 10.1038/sj.emboj.7601273 16917507 PMC1560355

[B2] Alimperti S. Andreadis S. T. (2015). CDH2 and CDH11 Act as Regulators of Stem Cell Fate Decisions. Stem Cell. Res. 14 (3), 270–282. 10.1016/j.scr.2015.02.002 25771201 PMC4439315

[B3] Amberger J. S. Hamosh A. (2017). Searching Online Mendelian Inheritance in Man (OMIM): A Knowledgebase of Human Genes and Genetic Phenotypes. Curr. Protoc. Bioinforma. 58, 1–12. 10.1002/cpbi.27 PMC5662200 28654725

[B4] Benitz W. E. Watterberg K. L. Cummings S. J. J. Eichenwald E. C. Goldsmith J. Poindexter B. B. (2016). Patent Ductus Arteriosus in Preterm Infants. Pediatrics 137 (1). 1. 10.1542/peds.2015-3730 26672023

[B5] Brohée S. Faust K. Lima-Mendez G. Vanderstocken G. van Helden J. (2008). Network Analysis Tools: from Biological Networks to Clusters and Pathways. Nat. Protoc. 3 (10), 1616–1629. 10.1038/nprot.2008.100 18802442

[B6] Cao Y. Wei M. Li B. Liu Y. Lu Y. Tang Z. (2016). Functional Role of Eukaryotic Translation Initiation Factor 4 Gamma 1 (EIF4G1) in NSCLC. Oncotarget 7 (17), 24242–24251. 10.18632/oncotarget.8168 27003362 PMC5029698

[B7] Chen S. Zhou Y. Chen Y. Gu J. (2018). Fastp: an Ultra-fast All-In-One FASTQ Preprocessor. Bioinformatics 34 (17), i884–i890. 10.1093/bioinformatics/bty560 30423086 PMC6129281

[B8] Clarke L. Zheng-Bradley X. Zheng-Bradley X. Smith R. Kulesha E. Xiao C. (2012). The 1000 Genomes Project: Data Management and Community Access. Nat. Methods 9 (5), 459–462. 10.1038/nmeth.1974 22543379 PMC3340611

[B9] Crockett S. L. Berger C. D. Shelton E. L. Reese J. (2019). Molecular and Mechanical Factors Contributing to Ductus Arteriosus Patency and Closure. Congenit. Heart Dis. 14 (1), 15–20. 10.1111/chd.12714 30468303 PMC6393200

[B10] Dobrikov M. Dobrikova E. Shveygert M. Gromeier M. (2011). Phosphorylation of Eukaryotic Translation Initiation Factor 4G1 (eIF4G1) by Protein Kinase Cα Regulates eIF4G1 Binding to Mnk1. Mol. Cell. Biol. 31 (14), 2947–2959. 10.1128/MCB.05589-11 21576361 PMC3133411

[B11] Erdogan F. Larsen L. A. Zhang L. Tumer Z. Tommerup N. Chen W. (2008). High Frequency of Submicroscopic Genomic Aberrations Detected by Tiling Path Array Comparative Genome Hybridisation in Patients with Isolated Congenital Heart Disease. J. Med. Genet. 45 (11), 704–709. 10.1136/jmg.2008.058776 18713793

[B12] Flanagan S. E. Patch A.-M. Ellard S. (2010). Using SIFT and PolyPhen to Predict Loss-Of-Function and Gain-Of-Function Mutations. Genet. Test. Mol. Biomarkers 14 (4), 533–537. 10.1089/gtmb.2010.0036 20642364

[B13] Forbes S. A. Bindal N. Bamford S. Cole C. Kok C. Y. Beare D. (2011). COSMIC: Mining Complete Cancer Genomes in the Catalogue of Somatic Mutations in Cancer. Nucleic Acids Res. 39 (Database issue), D945–D950. 10.1093/nar/gkq929 20952405 PMC3013785

[B14] Gene Ontology C. (2015). Gene Ontology Consortium: Going Forward. Nucleic Acids Res. 43, D1049–D1056. 10.1093/nar/gku1179 25428369 PMC4383973

[B15] Gong L.-C. Xu H.-M. Guo G.-L. Zhang T. Shi J.-W. Chang C. (2017). Long Non-coding RNA H19 Protects H9c2 Cells against Hypoxia-Induced Injury by Targeting MicroRNA-139. Cell. Physiol. Biochem. 44 (3), 857–869. 10.1159/000485354 29179202

[B16] Gravholt C. H. Viuff M. H. Brun S. Stochholm K. Andersen N. H. (2019). Turner Syndrome: Mechanisms and Management. Nat. Rev. Endocrinol. 15 (10), 601–614. 10.1038/s41574-019-0224-4 31213699

[B17] Groth K. A. Skakkebæk A. Høst C. Gravholt C. H. Bojesen A. (2013). Klinefelter Syndrome-A Clinical Update. J. Clin. Endocrinol. Metabolism 98 (1), 20–30. 10.1210/jc.2012-2382 23118429

[B18] Haimov O. Sehrawat U. Tamarkin-Ben Harush A. Bahat A. Uzonyi A. Will A. (2018). Dynamic Interaction of Eukaryotic Initiation Factor 4G1 (eIF4G1) with eIF4E and eIF1 Underlies Scanning-dependent and -Independent Translation. Mol. Cell. Biol. 38 (18). 1. 10.1128/MCB.00139-18 PMC6113598 29987188

[B19] Harakalova M. van der Smagt J. de Kovel C. G. F. Van't Slot R. Poot M. Nijman I. J. (2013). Incomplete Segregation of MYH11 Variants with Thoracic Aortic Aneurysms and Dissections and Patent Ductus Arteriosus. Eur. J. Hum. Genet. 21 (5), 487–493. 10.1038/ejhg.2012.206 22968129 PMC3641382

[B20] Haseeb A. Lefebvre V. (2019). The SOXE Transcription Factors-SOX8, SOX9 and SOX10-Share a Bi-partite Transactivation Mechanism. Nucleic Acids Res. 47 (13), 6917–6931. 10.1093/nar/gkz523 31194875 PMC6649842

[B21] Hoffman J. I. E. Kaplan S. (2002). The Incidence of Congenital Heart Disease. J. Am. Coll. Cardiol. 39 (12), 1890–1900. 10.1016/s0735-1097(02)01886-7 12084585

[B22] Kanehisa M. Furumichi M. Tanabe M. Sato Y. Morishima K. (2017). KEGG: New Perspectives on Genomes, Pathways, Diseases and Drugs. Nucleic Acids Res. 45 (D1), D353–D361. 10.1093/nar/gkw1092 27899662 PMC5210567

[B23] Karczewski K. J. Weisburd B. Thomas B. Solomonson M. Ruderfer D. M. Kavanagh D. (2017). The ExAC Browser: Displaying Reference Data Information from over 60 000 Exomes. Nucleic Acids Res. 45 (D1), D840–D845. 10.1093/nar/gkw971 27899611 PMC5210650

[B24] Landrum M. J. Chitipiralla S. Brown G. R. Chen C. Gu B. Hart J. (2020). ClinVar: Improvements to Accessing Data. Nucleic Acids Res. 48 (D1), D835–D844. 10.1093/nar/gkz972 31777943 PMC6943040

[B25] Leussis M. P. Berry-Scott E. M. Saito M. Jhuang H. de Haan G. Alkan O. (2013). The ANK3 Bipolar Disorder Gene Regulates Psychiatric-Related Behaviors that Are Modulated by Lithium and Stress. Biol. Psychiatry 73 (7), 683–690. 10.1016/j.biopsych.2012.10.016 23237312

[B26] Li B. Goyal J. Dhar S. Dimri G. Evron E. Sukumar S. (2001). CpG Methylation as a Basis for Breast Tumor-specific Loss of NES1/kallikrein 10 Expression. Cancer Res. 61 (21), 8014–8021. 11691827

[B27] Li N. Subrahmanyan L. Smith E. Yu X. Zaidi S. Choi M. (2016). Mutations in the Histone Modifier PRDM6 Are Associated with Isolated Nonsyndromic Patent Ductus Arteriosus. Am. J. Hum. Genet. 98 (6), 1082–1091. 10.1016/j.ajhg.2016.03.022 27181681 PMC4908195

[B28] Liang Y. Jiang L. Zhong X. Hochwald S. N. Wang Y. Huang L. (2019). Discovery of Aberrant Alteration of Genome in Colorectal Cancer by Exome Sequencing. Am. J. Med. Sci. 358 (5), 340–349. 10.1016/j.amjms.2019.07.012 31445671

[B29] Luo L.-Y. Meyts E. R.-D. Jung K. Diamandis E. P. (2001). Expression of the Normal Epithelial Cell-specific 1 (NES1; KLK10) Candidate Tumour Suppressor Gene in Normal and Malignant Testicular Tissue. Br. J. Cancer 85 (2), 220–224. 10.1054/bjoc.2001.1870 11461080 PMC2364047

[B30] Luo L. Herbrick J.-A. Scherer S. W. Beatty B. Squire J. Diamandis E. P. (1998). Structural Characterization and Mapping of the Normal Epithelial Cell-specific 1 Gene. Biochem. Biophysical Res. Commun. 247 (3), 580–586. 10.1006/bbrc.1998.8793 9647736

[B31] Masai I. Lele Z. Yamaguchi M. Komori A. Nakata A. Nishiwaki Y. (2003). N-cadherin Mediates Retinal Lamination, Maintenance of Forebrain Compartments and Patterning of Retinal Neurites. Development 130 (11), 2479–2494. 10.1242/dev.00465 12702661

[B32] Mayosi B. M. Fish M. Shaboodien G. Mastantuono E. Kraus S. Wieland T. (2017). Identification of Cadherin 2 ( CDH2 ) Mutations in Arrhythmogenic Right Ventricular Cardiomyopathy. Circ. Cardiovasc Genet. 10 (2). 1. 10.1161/CIRCGENETICS.116.001605 28280076

[B33] McKenna A. Hanna M. Banks E. Sivachenko A. Cibulskis K. Kernytsky A. (2010). The Genome Analysis Toolkit: a MapReduce Framework for Analyzing Next-Generation DNA Sequencing Data. Genome Res. 20 (9), 1297–1303. 10.1101/gr.107524.110 20644199 PMC2928508

[B34] Mitra S. Florez I. D. Tamayo M. E. Mbuagbaw L. Vanniyasingam T. Veroniki A. A. (2018). Association of Placebo, Indomethacin, Ibuprofen, and Acetaminophen with Closure of Hemodynamically Significant Patent Ductus Arteriosus in Preterm Infants. JAMA 319 (12), 1221–1238. 10.1001/jama.2018.1896 29584842 PMC5885871

[B35] Mohler P. J. Splawski I. Napolitano C. Bottelli G. Sharpe L. Timothy K. (2004). A Cardiac Arrhythmia Syndrome Caused by Loss of Ankyrin-B Function. Proc. Natl. Acad. Sci. U.S.A. 101 (24), 9137–9142. 10.1073/pnas.0402546101 15178757 PMC428486

[B36] Montero J. A. Giron B. Arrechedera H. Cheng Y. C. Scotting P. Chimal-Monroy J. (2002). Expression of Sox8, Sox9 and Sox10 in the Developing Valves and Autonomic Nerves of the Embryonic Heart. Mech. Dev. 118 (1-2), 199–202. 10.1016/s0925-4773(02)00249-6 12351187

[B37] O'Leary N. A. Wright M. W. Brister J. R. Ciufo S. Haddad D. McVeigh R. (2016). Reference Sequence (RefSeq) Database at NCBI: Current Status, Taxonomic Expansion, and Functional Annotation. Nucleic Acids Res. 44 (D1), D733–D745. 10.1093/nar/gkv1189 26553804 PMC4702849

[B38] O'Rahilly R. (1987). Human Embryo. Nature 329 (6138), 385. 10.1038/329385e0 3657957

[B39] Okonechnikov K. Conesa A. García-Alcalde F. (2016). Qualimap 2: Advanced Multi-Sample Quality Control for High-Throughput Sequencing Data. Bioinformatics 32 (2), btv566–294. 10.1093/bioinformatics/btv566 PMC4708105 26428292

[B40] Pannone L. Bocchinfuso G. Flex E. Rossi C. Baldassarre G. Lissewski C. (2017). Structural, Functional, and Clinical Characterization of a NovelPTPN11Mutation Cluster Underlying Noonan Syndrome. Hum. Mutat. 38 (4), 451–459. 10.1002/humu.23175 28074573

[B41] Radice G. L. Rayburn H. Matsunami H. Knudsen K. A. Takeichi M. Hynes R. O. (1997). Developmental Defects in Mouse Embryos Lacking N-Cadherin. Dev. Biol. 181 (1), 64–78. 10.1006/dbio.1996.8443 9015265

[B42] Richards S. Aziz N. Bale S. Bick D. Das S. Gastier-Foster J. (2015). Standards and Guidelines for the Interpretation of Sequence Variants: a Joint Consensus Recommendation of the American College of Medical Genetics and Genomics and the Association for Molecular Pathology. Genet. Med. 17 (5), 405–424. 10.1038/gim.2015.30 25741868 PMC4544753

[B43] Satoda M. Zhao F. Diaz G. A. Burn J. Goodship J. Davidson H. R. (2000). Mutations in TFAP2B Cause Char Syndrome, a Familial Form of Patent Ductus Arteriosus. Nat. Genet. 25 (1), 42–46. 10.1038/75578 10802654

[B44] Shang Y. Doan C. N. Arnold T. D. Lee S. Tang A. A. Reichardt L. F. (2013). Transcriptional Corepressors HIPK1 and HIPK2 Control Angiogenesis *via* TGF-β-TAK1-dependent Mechanism. PLoS Biol. 11 (4), e1001527. 10.1371/journal.pbio.1001527 23565059 PMC3614511

[B45] Steinhaus R. Proft S. Schuelke M. Cooper D. N. Schwarz J. M. Seelow D. (2021). MutationTaster2021. Nucleic Acids Res. 49 (W1), W446–W451. 10.1093/nar/gkab266 33893808 PMC8262698

[B46] Vanlerberghe C. Jourdain A.-S. Ghoumid J. Frenois F. Mezel A. Vaksmann G. (2019). Holt-oram Syndrome: Clinical and Molecular Description of 78 Patients with TBX5 Variants. Eur. J. Hum. Genet. 27 (3), 360–368. 10.1038/s41431-018-0303-3 30552424 PMC6460573

[B47] Wang K. Li M. Hakonarson H. (2010). ANNOVAR: Functional Annotation of Genetic Variants from High-Throughput Sequencing Data. Nucleic Acids Res. 38 (16), e164. 10.1093/nar/gkq603 20601685 PMC2938201

[B48] Wirgenes K. V. Tesli M. Inderhaug E. Athanasiu L. Agartz I. Melle I. (2014). ANK3 Gene Expression in Bipolar Disorder and Schizophrenia. Br. J. Psychiatry 205 (3), 244–245. 10.1192/bjp.bp.114.145433 24809399

[B49] Yang D. Liu B. C. Luo J. Huang T. X. Liu C. T. (2019). Kartagener Syndrome. QJM 112 (4), 297–298. 10.1093/qjmed/hcy242 30351433

